# Algebraic Representations of Entropy and Fixed-Sign Information Quantities

**DOI:** 10.3390/e27020151

**Published:** 2025-02-01

**Authors:** Keenan J. A. Down, Pedro A. M. Mediano

**Affiliations:** 1 Department of Psychology, School of Biological and Behavioural Sciences, Queen Mary University of London, Mile End Road, Bethnal Green, London E1 4NS, UK; 2 Department of Psychology, University of Cambridge, Downing Site, Downing Place, Cambridge CB2 3EB, UK; 3 Department of Computing, Imperial College London, 180 Queen’s Gate, South Kensington, London SW7 2RH, UK; p.mediano@imperial.ac.uk; 4 Division of Psychology and Language Sciences, University College London, 26 Bedford Way, London WC1H 0AP, UK

**Keywords:** information decomposition, co-information, entropy inequalities

## Abstract

Many information-theoretic quantities have corresponding representations in terms of sets. Many of these information quantities do not have a fixed sign—for example, the co-information can be both positive and negative. In previous work, we presented a signed measure space for entropy where the smallest sets (called atoms) all have fixed signs. In the present work, we demonstrate that these atoms have natural algebraic behaviour which can be expressed in terms of ideals (characterised here as upper sets), and we show that this behaviour allows us to make bounding arguments and describe many fixed-sign information quantity expressions. As an application, we give an algebraic proof that the only completely synergistic system of three finite variables *X*, *Y* and Z=f(X,Y) is the XOR gate.

## 1. Introduction

### 1.1. Information Decomposition

The Shannon entropy has many properties which intuitively mirror many set-theoretic identities. The *I*-measure of Yeung, built on earlier work by Hu Kuo Ting, fleshed out the correspondence between expressions of information quantities and set-theoretic expressions via a formal symbolic substitution [1,2]. Occasionally, however, the *I*-measure can be seen to conflate qualitatively different behaviours. A classic example of two such systems are the dyadic and triadic systems of James and Crutchfield [3], whose co-information signatures are identical, despite having qualitatively different constructions. In that work, they note that ‘no standard Shannon-like information measure, and exceedingly few nonstandard methods, can distinguish the two’.

One approach for discerning between these two systems is Partial Information Decomposition (PID), which aims to decompose the mutual information between a series of random source variables $X_{1},\ldots ,X_{n}$ and a target variable *T* into parts [4,5,6,7,8,9]. These parts are combinations of redundant, unique, and synergistic contributions to $I(X_{1},\ldots ,X_{n};T)$—which are all qualitatively distinct. The last of these pieces, the synergistic information, is information that is provided by multiple sources considered together but no source alone. For example, the perception of depth is severely hindered unless both eyes are recruited, and hence depth information is conferred synergistically. The co-information between three variables is, under the PID framework, the redundant (shared) information minus the synergistic information. For this reason, negative co-information is evidence that the system is exhibiting synergistic behaviour.

Beloved amongst the practitioners of PID is the XOR gate—one of the few examples of synergy on which there is nearly unanimous agreement. We will show in this work that the XOR gate is in fact the only system of three variables X,Y and Z=f(X,Y) which can have purely synergistic behaviour.

To accomplish this, we will leverage our previously introduced refined signed measure space ΔΩ, representing a collection of ‘atomic’ pieces of information [10,11]. This decomposition, built from the entropy loss when merging variable outcomes into coarser events (see for instance [12] for more context), allowed us to construct a Shannon-like measure which can discern between the dyadic and triadic systems of James and Crutchfield [3], which was demonstrated in [11]. This decomposition, coupled with the investigation of its algebraic properties in the present work, leads us to the capstone XOR result in Theorem 9.

### 1.2. Contributions

In the present work, we demonstrate that the constructed space ΔΩ has much natural algebraic behaviour when considered in tandem with the measure μ. We show that the structure of co-information, a standard information quantity [13], can be expressed algebraically inside of ΔΩ, and this description has very stable behaviour under the measure μ.

In Section 2, we present some background on the measure μ and recapitulate the main concepts introduced in [11]. From there, in Section 3, we develop the algebraic theory of this decomposition, introducing a new object, the ideal, and we show that it has some natural properties for simplifying representations of subsets in ΔΩ, highlighting how they can be used to generalise partitions in Ω.

After this, in Section 4, we show that the algebraic structure of these ideals plays uniquely well with the measure μ, allowing us to describe an algebraic property we call ‘strong fixed parity’, which, we show, corresponds to an information quantity having a fixed sign.

Lastly, in Section 5, we use these ideas to investigate the co-information between systems of variables, showing that this can be demonstrably fixed-sign in many cases. We finish with a result showing that the XOR gate is the only purely synergistic deterministic gate in three variables. That is, given three discrete variables *X* and *Y* with Z=f(X,Y) where *X* and *Y* are finite, the XOR is the only system with negative co-information for all probability mass functions on *X* and *Y*.

To start, we give a brief recapitulation of the concepts introduced in the previous work [11] here as background. Proofs for all new results can be found in Appendix A.

## 2. Background

### 2.1. Background on the Measure

In previous work, we introduced the signed measure space (ΔΩ,μ), where the σ-algebra is taken implicitly as the set of all subsets of ΔΩ. To start, we restate the definition of the space ΔΩ as was given in the first paper [10].

**Definition 1.**  *Let (Ω,F,P) be a finite probability space where the σ-algebra F is given by all subsets of *Ω*. Then, we define the* ***complex*** *(or content) of *Ω*, written ΔΩ, to be the simplicial complex on all outcomes ω∈Ω, with the vertices removed:*

(1) $$\Delta \Omega =\overset{N}{\underset{k=2}{⋃}}\Omega_{k}≅P\left(\Omega \right)\setminus \left(\{\{\omega \}:\omega \in \Omega \}\cup \{⌀\}\right)$$

*where $\Omega_{k}$ is the set of all subsets of size k inside of Ω.*

This space contains $2^{\left|\Omega \right|}-\left|\Omega \right|-1$ elements (called **atoms**) for a given finite outcome space Ω.

**Definition 2.**  *Given a discrete outcome space* Ω*, an* ***atom*** *is a subset S⊆Ω where |S|≥2.*

For a general set *S*, we use the notation $b_{S}$ for an atom, but where outcomes are explicitly labelled, e.g., S={1,2,3}, we might write $b_{\left\{1,2,3\right\}}$, $b_{123}$ or simply 123 where this is clear from context.

In order to construct the signed measure space (ΔΩ,μ), we must also define the measure. In the original work [10,11], this representation of the measure is given as a proposition. We give it here as the primary definition.

**Definition 3.**  *Let $T=\{p_{1},\ldots ,p_{k}\}$ be some subset of probabilities of an atom $\left\{\omega_{1},\ldots ,\omega_{n}\right\}$. For clarity, we write*

(2) $$\sigma \left(T\right)=\sigma \left(p_{1},\ldots ,p_{k}\right)={\left(p_{1}+\cdots +p_{k}\right)}^{\left(p_{1}+\cdots +p_{k}\right)}.$$

*Taking all subsets of the atom $\left\{\omega_{1},\ldots ,\omega_{n}\right\}$ of size k, we write*

(3) $$A_{k}=\prod_{\begin{array}{c} S\subseteq \{p_{1},\ldots ,p_{n}\}\\ |S|=k \end{array}}\sigma \left(S\right).$$

*Then the measure on the atom is given by*

(4) $$\mu \left(p_{1},\ldots ,p_{n}\right)=\sum_{k=1}^{n}{\left(-1\right)}^{n-k}\log\left(A_{k}\right).$$

This definition arises from the perspective of entropy loss, which has appeared previously in the literature and has some natural advantages over the classical formulation of entropy [12]. The measure given here is constructed using two steps: firstly, by considering the entropy loss *L* when a number of outcomes $\omega_{1},\ldots ,\omega_{t}$ are merged and treated as a single outcome; and secondly, by performing a Möbius inversion with *L* over the partially ordered set of subsets of Ω (ordered under inclusion).

Using the measure of loss *L* alone, while sufficient to derive a measure space (see [14]), does not possess sufficient resolution to capture all information quantities, missing quantities such as the mutual information and co-information. Incorporating the Möbius inversion breaks the construction into smaller pieces which multiple systems might share, creating an additive measure μ.

The entropy loss, as given immediately by a result of Baez et al. [12], is homogeneous of degree *d* when applied to the *d*-th Tsallis entropy [15]. By extension, the measure μ, which can be viewed as an alternating sum of the losses *L*, is also homogeneous of degree *d* when built on the *d*-th order Tsallis entropy. Moreover, the measure μ has some intriguing properties, which we shall briefly restate here. The interested reader should refer to the original works [10,11] for more detail.

**Example 1.**  *Let $\Omega =\{\omega_{1},\omega_{2},\omega_{3},\omega_{4}\}$ with corresponding probabilities $p_{1}=0.1$, $p_{2}=0.2$, $p_{3}=0.3$ and $p_{4}=0.4$. The atom $b_{12}$, which we might also write simply as 12 or {1,2}, has corresponding measure*

(5) $$\mu \left(\omega_{1},\omega_{2}\right)=\mu \left(0.1,0.2\right)=0.275\;\mathrm{bits}.$$

*The atom $b_{123}$, meanwhile, has a negative sign. Using the method given above, this is given by*

(6) $$\begin{array}{cc} \mu (\omega_{1},\omega_{2},\omega_{3}) & =\log_{2}\frac{{\left(0.1+0.2+0.3\right)}^{\left(0.1+0.2+0.3\right)}\cdot 0.1^{0.1}\cdot 0.2^{0.2}\cdot 0.3^{0.3}}{{\left(0.1+0.2\right)}^{\left(0.1+0.2\right)}\cdot {\left(0.1+0.3\right)}^{\left(0.1+0.3\right)}\cdot {\left(0.2+0.3\right)}^{\left(0.2+0.3\right)}} \end{array}$$

(7) $$\begin{array}{cc} \; & =\log_{2}\frac{0.6^{0.6}\cdot 0.1^{0.1}\cdot 0.2^{0.2}\cdot 0.3^{0.3}}{0.3^{0.3}\cdot 0.4^{0.4}\cdot 0.5^{0.5}}=-0.210\;\mathrm{bits}. \end{array}$$

*We will see in Theorem 1 that this change in sign is inevitable for certain atoms.*

**Lemma 1.**  *For $p_{1},\ldots ,p_{n},x\in R^{+}$ where n≥0, we have*

(8) $$\lim_{x\to 0}\mu \left(p_{1},\ldots ,p_{n},x\right)=0.$$

This lemma guarantees that the measure becomes null if any of the constituent probabilities are zero.

**Lemma 2.**  *Let $p_{1},\ldots ,p_{n-1},x\in R^{+}$ and let x vary. Then*

(9) $$\lim_{x\to \infty }|\mu \left(p_{1},\ldots ,p_{n-1},x\right)|=|\mu \left(p_{1},\ldots ,p_{n-1}\right)|.$$

This result shows that if one of the ‘probabilities’ tends to infinity, then the size of the entropy contribution tends towards that of an atom lying beneath it. Although discrete probabilities cannot tend to infinity, this result will prove useful for bounding arguments as part of Corollary 1 below.

Lastly, as a particularly intriguing property of the measure, its sign is known on all atoms of the partial order.

**Theorem 1.**  *Let $p_{2},\ldots ,p_{n}\in R^{+}$ be a sequence of nonzero arguments for n≥2 and m≥0. Then*

(10) $${\left(-1\right)}^{m+n}\frac{\partial^{m}\mu }{\partial x^{m}}\left(x,p_{2},\ldots ,p_{n}\right)\geq 0.$$

Setting m=0, it becomes clear that the sign of the measure μ on a given atom $\omega_{1},\ldots ,\omega_{n}$ is dependent only on the number of outcomes *n*. The co-information, by contrast, is not a fixed-sign quantity in general. For example, given three random variables, the co-information can be positive, negative, or even dependent on the underlying probabilities.

Coupled with Lemma 2, we have that μ varies monotonically between 0 and the magnitude of the atoms beneath it.

**Corollary 1**  (Magnitude can only decrease)**.** *Let $p_{1},\ldots ,p_{n-1},\tau \in R^{+}\cup \left\{0\right\}$ for n≥3. Then*

(11) $$|\mu \left(p_{1},\ldots ,p_{n-1},\tau \right)|<|\mu \left(p_{1},\ldots ,p_{n-1}\right)|.$$

This corollary is intriguing in that it bounds the contribution to the entropy of an atom by all of the atoms which lie under it in the partial order. This can be thought of as the notion that ‘higher-order contributions to the entropy are bounded above by lower-order contributions to the entropy’.

### 2.2. Ideals in Ring Theory

It may be helpful for some readers to briefly introduce the notion of an ideal as it appears in the algebraic theory of rings, since we will introduce an analogous object in the next section. While the *ideals* introduced in this work are constructs inside of a lattice (rather than a ring), they are usually first introduced inside of rings, where their structure is intuitive. In addition, there are ways in which it might be natural to extend the definition given in the remainder of this work to an ideal in a ring. Thus, we have chosen to use the name ‘ideal’ rather than ‘order ideal’ (as might be more standard). The reader familiar with the algebraic theory of rings and ideals can confidently skip this subsection.

A **ring** is, broadly speaking, a set where there exist notions of addition, subtraction, and multiplication (though not division, in general). A standard example is the integers Z or the ring of polynomials in a single variable *x* with real coefficients R[x].

**Definition 4**  (Ideal in a ring)**.** *An **ideal** I over a (commutative) ring R is a subset I⊆R such that I is a group under addition inside of I and closed under multiplication with an element of R. That is, for any x,y∈I and r∈R, we have*

(12) $$\begin{array}{cc} -x & \in I \end{array}$$

(13) $$\begin{array}{cc} x+y & \in I \end{array}$$

(14) $$\begin{array}{cc} rx & \in I. \end{array}$$

*Note that because of the first and second requirements, every ring ideal also contains zero.*

Ideals capture a notion of dependency between elements in the ring. The presence of one element in the ideal forces those ‘above’ the element to also be contained in the ideal (where the order can be described by multiplication/divisibility). Ideals also have some convenient properties.

**Proposition 1.**  *Let I,J be two ideals of a ring R. Then*

(15) $$\begin{array}{cc} I\cap J & =\{x\in R:\;x\in I\;\mathrm{and}\;x\in J\} \end{array}$$

(16) $$\begin{array}{cc} I+J & =\{x+y:\;x\in I,y\in J\} \end{array}$$

*are both ideals.*

A classic example of an ideal in Z is 〈n〉, which is the set of all numbers which are divisible by an integer *n*. If *a* and *b* are elements of 〈n〉 (i.e., they are both divisible by *n*), then we certainly must have that a+b∈〈n〉 (their sum is also divisible by *n*), and multiplying by *any* number r∈Z will force ar∈〈n〉, as the factor of *n* is still present.

Ideals also play a large role in algebraic geometry, where polynomial rings are a natural point of study. In this scenario, the ideal 〈f(x)〉⊆R[x] is the set of all polynomials which contain f(x) as a factor. Equivalently, it is the set of polynomials which, given that f(x)=0, must also be zero.

In much the same way that knowledge that *n* is divisible by 2 implies 3n is divisible by 2, knowledge that two outcomes $\omega_{1},\omega_{2}$ are distinct automatically provides knowledge that some pair inside of $\omega_{1},\omega_{2},\omega_{3}$ is distinct. This is the structure of dependency which we make use of when restating Definition 5 below.

## 3. An Algebraic Perspective on Entropy

### 3.1. Representing Information Quantities Inside ΔΩ

We briefly state a key result from our previous work [11], where we expressed the entropy associated to a random variable *X* in terms of a subset of ΔΩ.

**Definition 5.**  *Given a random variable X, we define the* ***content*** *ΔX inside of ΔΩ to be the set of all atoms inside of ΔΩ crossing a boundary in X. That is, if X corresponds to a partition $P_{1},\ldots ,P_{n}$, then*

(17) $$\Delta X=\{b_{S}:S\subseteq \Omega ,\exists \;\omega_{i},\omega_{j}\in S\;\mathrm{with}\;\omega_{i}\in P_{k},\;\omega_{j}\in P_{l}\;\mathrm{such}\;\mathrm{that}\;k\neq l\;\}.$$

*Intuitively, this means that at least two of the outcomes in the atom $b_{\omega_{1}\ldots \omega_{n}}$ correspond to distinct events in X, although possibly more. We will in general make use of *Δ* to represent the logarithmic decomposition functor from random variables and information quantities to their corresponding sets in ΔΩ. Note that we often write 123 to refer to $b_{1,2,3}$ for added readability.*

As expected, we have that μ(ΔX)=H(X), and we concretise this in a theorem, which is taken from [11].

**Theorem 2.**  *Let R be a region on an I-diagram of variables $X_{1},\ldots ,X_{r}$ with Yeung’s I-measure. In particular, R is given by some set-theoretic expression in terms of the set variables ${\tilde{X}}_{1},\ldots ,{\tilde{X}}_{r}$ under some combination of unions, intersections and set differences.*

*Making the formal substitution*

(18) $${\tilde{X}}_{1},{\tilde{X}}_{2},\ldots ,{\tilde{X}}_{r}\;⟷\;\Delta X_{1},\Delta X_{2},\ldots ,\Delta X_{r}$$

*to obtain an expression ΔR, the content corresponding to the region R of the I-diagram, in terms of the $\Delta X_{i}$, we have*

(19) $$I\left(R\right)=\sum_{B\in \Delta R}\mu \left(B\right).$$

*That is, the interior loss measure μ is consistent with Yeung’s I-measure.*

For examples on how this measure can be interpreted geometrically, as well as all proofs of the above results, we refer the interested reader to the previous work [11], where we present figures and diagrams demonstrating the geometric and set-theoretic significance of the atoms of our construction.

Many questions about the underlying structure of this space remain to be answered. One peculiarity is that most atoms do not normally appear alone in information quantities. For example, given that an atom $\omega_{1}\ldots \omega_{n}\in \Delta X$ appears in a content, we must also have that the atom $\omega_{1}\ldots \omega_{n}\omega_{n+1}\in \Delta X$ appears in the same content, as the definition is just those atoms which, as a set, cross a boundary in *X*. While all atoms have an interpretation of crossing boundaries in partitions, individual atoms, at first, do not seem to have much meaning without other atoms in context. Understanding the structural interrelationship between all atoms would allow for a better understanding of the relationship between different information measures.

In the rest of this section, we explore the structure of our decomposition in the language of posets and **upper sets** (or **ideals**) on those posets, which appear to provide the natural language for the analogous ‘molecules’ to our atoms. We begin by defining an order ≼ on our atoms before giving a definition for **ideals** in ΔΩ. From there, we will show that all co-information expressions correspond to ideals and vice versa. We finish this section by characterising the ideals which correspond to the entropy of a variable.

### 3.2. Ideals in ΔΩ

**Definition 6.**  *Let $b_{S_{1}},b_{S_{2}}\in \Delta \Omega$ where $S_{1},S_{2}\subseteq \Omega$. We define a partial ordering on the set ΔΩ by setting $b_{S_{1}}≼b_{S_{2}}$ whenever $S_{1}\subseteq S_{2}$.*

The following definition is taken from [16].

**Definition 7.**  *Given a (partially) ordered set P, a subset J⊆P is called an* ***order ideal*** *(* ***upper-set*** *,* ***up-set*** *,* ***increasing set*** *) if, for all x∈J and y∈P, we must have y∈J whenever x≼y and J≠⌀. That is, J is non-empty and closed under ascending order.*

*Following standard language, we will say that an ideal J is* ***generated*** *by a collection of elements $g_{1},\ldots ,g_{t}$ if, for all b∈J, we have $g_{i}≼b$ for at least one $g_{i}\in \left\{g_{1},\ldots ,g_{t}\right\}$. We will write $J=\langle g_{1},\ldots ,g_{t}\rangle$.*

*That is to say, the ideal J is the set in ΔΩ which contains $g_{1},\ldots ,g_{t}$ and all elements which lie above them in the order.*

We note that we deviate from standard nomenclature in this case and refer to these upper sets simply as ‘ideals’. In classical order theory, ideals in lattices are down sets rather than upper sets and are subject to an additional constraint. In the current work, we use **ideal** in the order-ideal sense, as we expect that future work on ΔΩ as a ring might make this definition more intuitive.

We will concern ourselves later with the relationship between the generators of an ideal and the measure of the ideal itself. The following definition for the **degree** of an atom is taken from [11], which we then extend to ideals.

**Definition 8.**  *Let $b=\omega_{1}\ldots \omega_{d}\in \Delta \Omega$. We define the* ***degree*** *of b to be the number of outcomes it contains. That is, deg(b)=d.*

**Definition 9.**  *We will call J a* ***degree*** *n* ***ideal*** *or* *n-**ideal*** *if it can be generated by purely degree n atoms.*

One significant motivation for introducing the language of ideals is to simplify the description of the sets constructed by the decomposition. Rather than writing out the complete set of all atoms, it is often possible to write out the generators of the set as an ideal, vastly reducing the complexity of the notation. Much like ideals in ring theory, it is straightforward to describe the intersection and union of ideals using the generators alone. We introduce some notation and give a proposition to this effect.

**Notation 1.**  *To further the parallel to ideals in rings, it is sometimes useful to introduce multiplicative notation for the union of two atoms. That is, we will make use of the notation*

(20) $$b_{S\cup T}=b_{S}b_{T}.$$

*For example, using the shorthand notation from before, we have 123·234=1234.*

**Proposition 2.**  *Let $G=\{g_{1},\ldots ,g_{n}\}$ be a set of generators for the ideal $I=\langle G\rangle =\langle g_{1},\ldots ,g_{n}\rangle$, and let $H=\{h_{1},\ldots ,h_{m}\}$ be a set of generators for the ideal $J=\langle H\rangle =\langle h_{1},\ldots ,h_{m}\rangle$, where I and J are ideals inside ΔΩ. Then,*

(21) $$I\cup J=\langle g_{1},\ldots ,g_{n},h_{1},\ldots ,h_{m}\rangle =\langle \;b|b\in G\cup H\;\rangle ,$$

(22) $$I\cap J=\langle g_{1}h_{1},g_{1}h_{2},\ldots ,g_{n}h_{m-1},g_{n}h_{m}\rangle =\langle \;gh|g\in G,h\in H\;\rangle$$

*This formulation mimics the natural behavior of ideals in rings.*

**Remark 1.**  *It is occasionally convenient for notation to consider ideals generated by single outcomes ω even though we formerly excluded these and *⌀* from ΔΩ. We may alternate between including and excluding these atoms for algebraic simplicity. Recall that the singlet and empty atoms do not contribute to the entropy, so this choice of notation does not affect the measure (we note also that including these entities would endow ΔΩ with the complete structure of a lattice, which, although currently not required, might be useful for future work).*

**Example 2.**  *Let Ω={1,2,3,4}. Then ΔΩ={12,13,14,23,24,34,123,124,134,234,1234}. Inside of ΔΩ, an ideal consists of all atoms which ‘contain’ a generator. For example, the ideal 〈12,13〉={12,13,123,124,134,1234} is a 2-atom ideal, as it is generated by degree 2 atoms. All of the atoms in this ideal must contain either outcomes 1 and 2 or outcomes 1 and 3, or both.*

### 3.3. Representation of Quantities with Ideals

We should justify that these ideals are a natural object of study. As it turns out, all entropy expressions without multiplicity and without conditioning are given by ideals, which follows from the next lemma.

**Lemma 3.**  *Given any co-information $I(X_{1};\ldots ;X_{t})$ (including entropy and mutual information), the corresponding content $\Delta X_{1}\cap \cdots \cap \Delta X_{t}$ is an ideal.*

**Figure 1:**
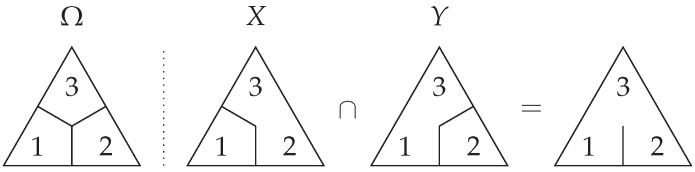
An outcome space Ω={1,2,3} and two variables *X* and *Y* defined over Ω. In this case, the intersection of the contents ΔX∩ΔY is given by the ideal 〈12〉. That is to say, I(X;Y)=μ(〈12〉). If the mutual information could be represented by a partition in this case, we would obtain something like the above intersection. This is, of course, impossible in the language of partitions but valid in ideals.

**Example 3.**  *It is worth noting that ideals themselves do not, in general, have corresponding partitions, but every partition has a corresponding ideal. As an example for how to conceptualise these ‘sub-partitions,’ consider the system Ω={1,2,3} where X has partition {{1},{2,3}} and Y has partition {{1,3},{2}} as per Figure 1.*

*We have that*

(23) $$\begin{array}{cc} \Delta X & =\{12,13,123\}\;\mathrm{and} \end{array}$$

(24) $$\begin{array}{cc} \Delta Y & =\{12,23,123\}. \end{array}$$

*Taking the mutual information between these sets corresponds algebraically to the intersection ΔX∩ΔY=〈12,13〉∩〈12,23〉=〈12〉={12,123}.*

*We note that this upper set 〈12〉 corresponds to the ability to discern between 1 and 2, but not between 1 and 3, or 2 and 3. Moreover, the upper set 〈12〉, despite not representing a partition itself, gives the mutual information when measured, i.e., μ(〈12〉)=I(X;Y).*

*That is to say, generalising from the language of partitions to the language of ideals has allowed us to properly describe mutual information—a quantity which partitions cannot in general represent.*

As it turns out, the converse to Lemma 3 also holds, which we state here.

**Theorem 3.**  *Let* Ω *be a finite outcome space and let $\left\{X_{a}:a\in A\right\}$ be the collection of all possible random variables defined on* Ω *(indexed by A). Then, there is a one-to-one correspondence*

(25) {idealsinΔΩ}↔{possibleco-informationsonΩ}

*where the co-informations are given by $I(X_{1};\ldots ;X_{j})$ for any number of arbitrary variables $X_{1},\ldots ,X_{j}$ defined on* Ω.

This result tells us that for any valid co-information on some collection of variables defined on an outcome space Ω, then there is a corresponding ideal in ΔΩ, and for every ideal in ΔΩ, there is a corresponding collection of variables which give the resulting co-information. As an immediate side effect of this result, we have an alternative derivation of proposition 33 in [11]:

**Corollary 2.**  *Let *Ω* be a finite outcome space. Then, there is a one-to-one correspondence*

(26) $$\left\{\;\mathrm{subsets}\;\mathrm{of}\;\Delta \Omega \;\right\}↔\left\{\;\mathrm{entropy}\;\mathrm{expressions}\;\mathrm{without}\;\mathrm{multiplicity}\;\mathrm{on}\;\Omega \;\right\},$$

*where by an ‘entropy expression without multiplicity’ we mean an expression of the form*

(27) $$\sum_{P\;\mathrm{partitioning}\;\Omega }n_{P}H\left(P\right)$$

*for $n_{P}\in Z$ where no region is double-counted in any I-diagram.*

Note that for the purposes of these two results, we do not consider the singlet atoms {ω} or the empty set to be elements of ΔΩ, as they contribute no entropy (see [11] for more justification).

This result shows that with clever inclusion and exclusion, it is always possible to extract individual atoms as classical entropy expressions on variables in ΔΩ. That is, they form a natural basis for entropy expressions. As such, the atoms of ΔΩ are uniquely placed for a module-theoretic or vector-space perspective on information.

Since these atoms appear to be a natural basis for entropy expressions, if we count them without multiplicity, we are able to determine how many expressions for information exist without accounting for the same contribution multiple times. We give a corollary to this end.

**Corollary 3.**  *Given a finite outcome space *Ω* with |Ω|=n, there are $2^{2^{n}-n-1}$ possible classical entropy expressions without multiplicity.*

Counting with multiplicity, we can see that the space of all entropy expressions on Ω is a free module over Z, where the atoms *b* form a very natural basis.

We now state a practical result which tells us, intuitively, exactly which ideals correspond to partitions and how we can find the generators of the ideal corresponding to a finite random variable *X*. For the purpose of this result, it is again useful to consider the singlets {ω}, but the resulting representation of ΔX will not contain them.

**Theorem 4.**  *Let X be a discrete random variable on the outcome space *Ω*, where X has corresponding partition $Q_{t}:t\in T$ for some indexing set T of parts $Q_{t}$. Then ΔX as an ideal is given by*

(28) $$\Delta X=\underset{\begin{array}{c} a,\;b\;\in \;T\\ a\;\neq \;b \end{array}}{⋃}\langle \left\{\omega \in Q_{a}\right\}\rangle \cap \langle \left\{\omega \in Q_{b}\right\}\rangle .$$

*We note in particular that in posets, the union of order ideals is equal to the order ideal with the union of their generators. Equivalently, we have*

(29) $$\Delta X=\underset{t\;\in \;T}{⋃}\;\langle \left\{\omega \in Q_{t}\right\}\rangle \cap \langle \left\{\omega \in Q_{t}^{c}\right\}\rangle =\underset{t\;\in \;T}{⋃}\;\langle \left\{\omega \bar{\omega }:\omega \in Q_{t},\bar{\omega }\in Q_{t}^{c}\right\}\rangle .$$

*In particular, ΔX as an ideal is generated by 2-atoms.*

**Example 4.**  *Consider the outcome space Ω={1,2,3,4}. Now, let X be the variable with partition {{1,2},{3},{4}}. Then, as an ideal, we have*

(30) $$\begin{array}{cc} \Delta X & =(\langle 1,2\rangle \cap \langle 3\rangle )\cup (\langle 1,2\rangle \cap \langle 4\rangle )\cup (\langle 3\rangle \cap \langle 4\rangle )\\ \; & =\langle 13,23\rangle \cup \langle 14,24\rangle \cup \langle 34\rangle \\ \; & =\langle 13,23,14,24,34\rangle . \end{array}$$

**Corollary 4.**  *Let $X_{a},a\in A$ be a family of discrete variables on the outcome space *Ω*. Knowledge of how the 2-atoms are located among the $\Delta X_{a}$ is sufficient to describe how all other atoms are located.*

Restating this, knowledge of the 2-atoms contained in each $\Delta X_{a}$ is sufficient to deduce the presence of any atom in any set-theoretic expression constructed using the $\Delta X_{a}$.

We have now successfully described the structure of entropy through the algebraic lens of ideals in a poset and illustrated that ideals in this lattice correspond to co-informations, while other subsets of ΔΩ correspond to entropy expressions on Ω.

To illuminate the power of this flavour of the theory, in the next section, we shall see how these ideals interact with the measure μ and use our results to demonstrate that mutual information is always given by a degree 2 ideal. Not only this, but we will give a generalisation which bounds the degree of the generators for ideals representing the intersection of more than two variables. We then extend these techniques to explore ideals giving fixed-sign information quantities. This intriguing result will show that a surprising amount can be learned about an information quantity without much knowledge of the underlying probabilities.

## 4. Properties of the Measure on Ideals

We have now developed lots of language for discussing the ideals inside of the lattice ΔΩ. Moreover, having seen that co-information is perfectly described by these ideals, it would be a natural question to ask how the measure μ interacts with the ideal structure. In this section, we will demonstrate that the entropy contribution of an ideal can, much like atoms, be neatly categorised as either positive or negative in many cases, and we shall see that this provides various tools for constructing new bounds.

In this section, we begin by demonstrating that the mutual information is always given by a degree 2 ideal. To accomplish this, we shall need the following notion of *restriction*, which we shall utilise in the proofs to follow.

**Definition 10.**  *Let X be a random variable on a finite outcome space *Ω*, and let S⊆Ω. We define the* ***restriction*** *of a collection of atoms W⊆ΔΩ to S as*

(31) $$W_{S}=\left\{b_{Q}\in W:Q\subseteq S\right\}.$$

*In particular, we will use the notation $\Delta X_{S}$ and ${\langle \ldots \rangle }_{S}$ or occasionally ${\cdot |}_{S}$ to construct contents and ideals inside of restrictions.*

Restriction simply allows us to focus our attention on a subset of the atoms—in particular, those whose outcomes all belong to the restricting subset *S*. Note that given some subset W⊆ΔΩ, we have that $W_{S}\subseteq W$ rather than operating with an entirely new class of atoms.

One of the strengths of the measure μ beyond entropy alone is that μ is homogeneous and works across multiple scales. As such, every statement and piece of structure given here for ΔΩ and ideals therein *also* applies to $\Delta \Omega_{S}$ for S⊆Ω. We shall demonstrate that many problems exploring the intersection of ideals (and hence the intersection of entropies) can be much simplified by restricting. We proceed with the first result on mutual information, where we use this concept in the proof.

**Theorem 5**  (Mutual information is a degree 2 ideal). *Let X and Y be two random variables. Then, there exists a set of 2-atom generators $\left\{a_{i}b_{i}:a_{i},b_{i}\in \Omega \right\}$ for i=1,…,k such that*

(32) $$I\left(X;Y\right)=\mu (\langle a_{1}b_{1},\ldots ,a_{k}b_{k}\rangle )$$

We have demonstrated something rather intriguing: mutual information looks a lot like a normal variable content in that it is always generated by degree 2 atoms, but the generators of mutual information do not need to correspond to a representable subset of ΔΩ. When working with ideals in general, one would expect that the intersection between generators of degree *m* and *n* would have degree bounded above by m+n, so it is rather surprising that the mutual information has this property.

Extending the investigation of the Gács–Körner common information in [11], the following can now be seen:

**Corollary 5.**  *The Gács-Körner common information is generated by degree 2 atoms, and the generating set is the largest subset of generators of the mutual information, which is representable by some random variable (in [10] this property is referred to as ‘discernibility’).*

This result confirms our natural intuition for selecting generators to construct a variable. We provide also a generalisation of Theorem 5 to co-information.

**Theorem 6.**  *Let *Ω* be the joint outcome space of M discrete variables $X_{1},\ldots ,X_{M}$. Then, the content of $I(X_{1};\ldots ;X_{M})$ can be completely generated by atoms of degree at most M.*

This result states that the degree of the generators of an ideal corresponding to some co-information is always bounded above by the number of variables. This result vastly reduces the search space of generators when studying the properties of co-information, and we make use of it in our study of fixed-parity systems in the next section.

**Example 5.**  *Consider the standard OR gate given by outcomes (X,Y,Z=OR(X,Y)), which we label as follows:*

*X*

*Y*


Z=OR(X,Y)


*Outcome (ω)*

*0*

*0*

*0*

*1*

*0*

*1*

*1*

*2*

*1*

*0*

*1*

*3*

*1*

*1*

*1*

*4*

*Note that in this instance, we have*

**Figure 2:**
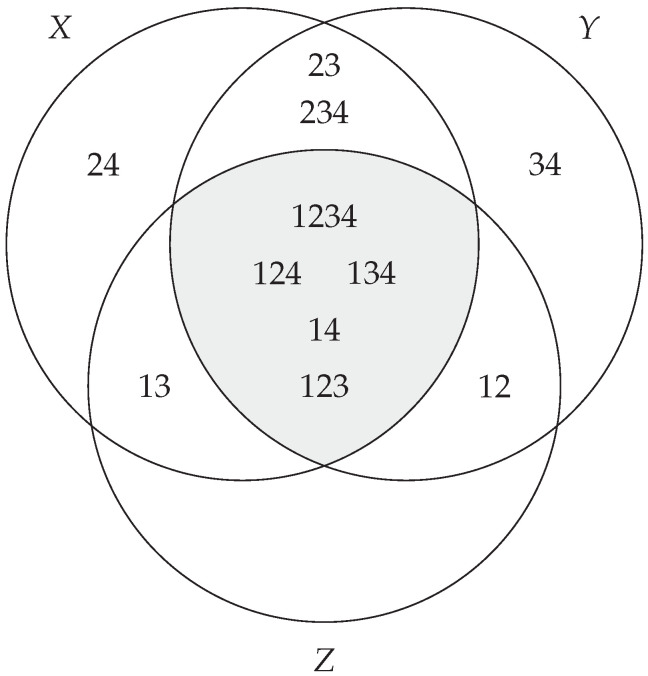
An *I*-diagram demonstrating the entropy structure for the OR gate. The shaded region corresponds to the ideal 〈14,123〉. Note that in this case, the degree of the generators is bounded above by 3, as we have the intersection of 3 variables as per Theorem 6.

(33) I(X;Y;Z)=μ(〈14,123〉),

*which is generated by at most degree 3 atoms, as expected. Note that we are discussing the structure of the OR gate without any mention of the probabilities. A diagram representing the structure is given in Figure 2. As expected, the degree of the generators is bounded above by 3.*

Although this is an interesting representation of the structure of the co-information between random variables, we have not said much yet about the relationship between these ideals and their measures μ(J). As it turns out, for certain classes of ideals, the sign of μ(J) is just as easy to characterise as the signs of the atoms themselves.

**Lemma 4.**  *Let $J=\langle \omega_{1}\cdots \omega_{d}\rangle$ be an ideal generated by a single degree d atom with* *$P\left(\omega_{1}\right),\ldots ,P\left(\omega_{d}\right)\neq 0$. Then, ${\left(-1\right)}^{d}\mu \left(J\right)>0.$*

This result is quite powerful, as it tells us that in certain scenarios, we can know the sign of the ideal and the information measure it represents without any knowledge of the probabilities. We will strengthen this result shortly to demonstrate that certain classes of ideals, which we call strongly fixed-parity ideals, have fixed-sign measures.

**Definition 11.**  *Let $J=\langle g_{1},\ldots ,g_{j}\rangle$ be an ideal. If j=1, we say that J is* ***strongly fixed parity**, and set the* ***parity*** *of J as $P\left(J\right)={\left(-1\right)}^{\deg g_{1}}.$*

*Moreover, if j≥2, we shall say that J has* ***strongly fixed parity*** *if there is an expression*

(34) $$\mu \left(J\right)=P\left(J\right)\sum_{\alpha \in A}P\left(J_{\alpha }\right)\mu \left(J_{\alpha }\right)$$

*for some finite collection of fixed-parity ideals $\left\{J_{\alpha }:\alpha \in A\right\}$, with the equality holding across all probability distributions P on *Ω* and some P(J)∈{−1,1}, which we call the* ***parity*** *of J*.

*Lastly, we shall say that an ideal J⊆ΔΩ is of* ***strongly mixed parity*** *if it has generators of both even and odd degree.*

**Example 6.**  *The ideal 〈12,23〉 is strongly fixed even parity as*

(35) $$\begin{array}{cc} \mu (\langle 12,23\rangle ) & =\mu (\langle 12\rangle )+\mu (\langle 23\rangle )-\mu (\langle 12\rangle \cap \langle 23\rangle )\\ \; & =\mu (\langle 12\rangle )+\mu (\langle 23\rangle )-\mu (\langle 123\rangle ). \end{array}$$

*The ideal 〈123〉 has strong negative (odd) parity, and the two degree 2 ideals have strong positive (even) parity. When composing the parities, the measure of the whole set is given by three positive parts, so it makes sense to call 〈12,23〉 positive fixed-parity.*

**Theorem 7**  (Ideals of Strong Parity)**.** *Let J be an ideal in ΔΩ. If J is of strongly even parity, then μ(J)≥0, and if J is of strongly odd parity, then μ(J)≤0.*

This result is most pleasant as it reflects what feels like a natural intuition for how these systems should behave. In particular, given a system of variables $X_{1},\ldots ,X_{n}$, defined by partitions on a finite outcome space Ω, information quantities which reflect strongly fixed-parity ideals have a predetermined sign for any underlying probability distribution over Ω.

In this section, we demonstrated that the algebraic construction of ideals in our poset from the previous section plays remarkably well with the measure μ of our construction, and we developed several tricks for manipulating expressions in ΔΩ. We build on this theory in the next section and apply it to the problem of finding purely synergistic systems of the form *X*, *Y*, and Z=f(X,Y).

## 5. Fixed-Parity Systems

To motivate our investigation, we give here an example of how certain information quantities do not have fixed sign.

**Example 7.**  *Let X and Y be binary variables with Z=OR(X,Y).*

*Recall that the co-information (also known as the interaction information [13,17]) is given by*

(36) $$\begin{array}{cc} I(X;Y;Z)= & H(X)+H(Y)+H(Z)\\ \; & -H(X,Y)-H(X,Z)-H(Y,Z)\\ \; & +H(X,Y,Z). \end{array}$$

*In the case that P(X=x,Y=y)=0.25 for all outcomes (x,y)∈Ω, we have that the co-information is I(X;Y;Z)≈−0.19 bits, being negative in this case. However, in the case that P(X=0,Y=0)=P(X=1,Y=1)=0.45 and P(X=0,Y=1)=P(X=1,Y=0)=0.05, then we have I(X;Y;Z)≈0.52 bits. That is, for this system (and many others), knowledge of the structure of the outcomes alone (that is, any prior knowledge that certain combinations of symbols have zero probability) is not sufficient to determine the sign of the co-information, and it depends upon the underlying probabilities of the system states. We shall see why this is the case for the OR gate in particular in this section.*

We now give a definition to connect the algebraic perspective on ideals to information quantities.

**Definition 12.**  *Let $X_{1},\ldots ,X_{n}$ be a system of variables on a finite outcome space *Ω*. We say that the system $X_{1},\ldots ,X_{n}$ is a* ***fixed sign*** *or* ***fixed parity*** *system if the sign of*

(37) $$I(X_{1};\ldots ;X_{n})$$

*is fixed regardless of the underlying probability distribution. Similarly, we say that an entropy expression $E(X_{1},\ldots ,X_{n})$ defined on *Ω* has* ***fixed parity*** *if its sign is always fixed regardless of the underlying probabilities in $X_{1},\ldots ,X_{n}$. We say that the system is* ***negative/odd fixed parity*** *if the co-information is always negative, and it is* ***positive/even fixed parity*** *if the co-information is always positive.*

There is a natural dual question to be asked here: is it possible to have a fixed-parity system where the co-information is a strongly-mixed ideal—that is, generated by elements of varying degrees? The next theorem shows this is, in fact, impossible.

**Theorem 8.**  *A system of variables $X_{1},\ldots ,X_{n}$ with $I(X_{1};\ldots ;X_{n})$ given by an ideal of strongly mixed-parity cannot have a fixed parity.*

*That is to say, being strongly-mixed (an algebraic property) implies mixed signs (a property of the measure on the set).*

This theorem gives a partial converse to Theorem 7 in that it gives us a way to characterise some mixed-parity systems. Although we have not characterised every fixed-parity or mixed-parity system, we expect such a characterisation in terms of ideal properties (algebraic reasoning alone) should be possible. However, the tools we have already constructed are sufficient to show the main result of this paper.

**Theorem 9.**  *The only negative fixed-parity (always synergy-dominated) system given by two finite variables X,Y and a deterministic function Z=f(X,Y) is the XOR gate.*

**Figure 3:**
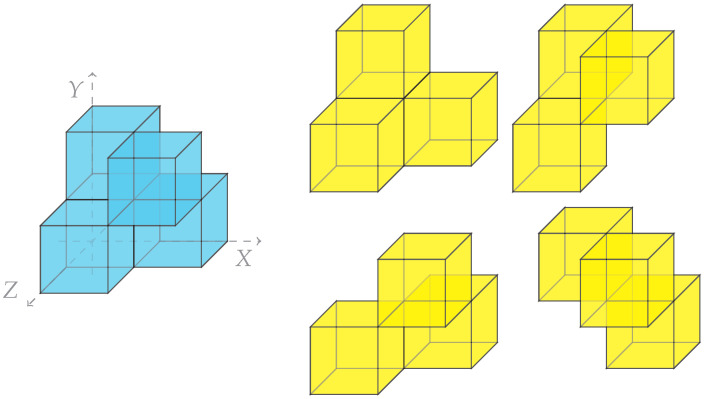
The XOR gate and the four subsets of outcomes which directly contribute to the negativity of the co-information. The presence of nonzero probabilities in one of these ‘flower-shaped’ patterns is required for any synergistic effect in a system of three binary outcomes.

Of note is that the XOR gate is generated by the presence of three degree 3 atoms, as depicted in Figure 3. Each of these atoms generates the synergistic effect. While they all exhibit a change in *X*, *Y* and *Z*, the places where these changes are seen are distinct between the variables. There is no single degree 2 atom $\omega_{1}\omega_{2}$ (the knowledge that $\omega_{1}$ and $\omega_{2}$ are distinct) which the three variables share.

## 6. Discussion

In this paper, we extended our results on the measure μ from [10,11] to an algebraic construction inside of the space ΔΩ. We demonstrated that in many cases, the study of ‘ideals’ (in the order-theoretic sense) inside of ΔΩ simplifies bounding problems, and we showed that these ideals form a natural intermediate language between partitions and have useful behaviour in tandem with the measure μ.

While in the present work, the issue of bounding is applied to the study of fixed-sign quantities, we expect these techniques can be used in multiple scenarios where bounding over all possible probabilities is required. Moreover, we expect that there is a stronger version of Theorem 7 which might be stated with weaker restrictions on the underlying ideals, and a full characterisation of all fixed-parity systems would be an insightful direction. Future work may develop this theory.

One particularly intriguing result given here is that the underlying cause of three-variable synergy appears to be easily characterisable by its geometry alone. The three-outcome ‘flower’ shape presented in Figure 3 is required for the existence of synergy in three variables, being the only generator of three-variable co-information which has a negative measure, with an intrinsically three-dimensional shape. We leave here the problem of classifying the generators of various orders open. While it is straightforward to see all such effects now for two or three variables, the case beyond three variables appears to be more opaque. In particular, we expect that there may be vastly many new information effects in four or more variables which cannot geometrically exist in simpler systems.

We applied the new algebraic results developed in this paper to show that the XOR gate is the only purely synergistic system (i.e., always possessing negative co-information) of finite variables X,Y and Z=f(X,Y) for a deterministic function *f* (see also [18]). In particular, we highlight that this was achieved with a purely algebraic proof that did not require any navigation of the space of probability distributions.

We hope this work might be applied to the problem of Partial Information Decomposition (PID) [3,5,6,8,19] and contribute to the widening body of knowledge in set-theoretic information theory.

## Data Availability

The original contributions presented in this study are included in the article. Further inquiries can be directed to the corresponding author.

## Appendix A. Proofs for Results

The proof for Theorem 2 and results in Section 2 can be found in [11], where we also give an alternative expression for Definition 3.

**Proof of Proposition 2.** Suppose b∈I∪J. Then, either b≽g or b≽h for some g∈G or h∈H. Hence, $b\in \langle g_{1},\ldots ,g_{n},h_{1},\ldots ,h_{m}\rangle$ as needed. Conversely, if *b* is contained in $\langle g_{1},\ldots ,g_{n},h_{1},\ldots ,h_{m}\rangle$, then $b≽b^{\prime }$ for some $b^{\prime }\in \left\{g_{1},\ldots ,g_{n},h_{1},\ldots ,h_{m}\right\}$, so *b* must be contained in either *I* or *J*, so b∈I∪J. Suppose that b∈I and b∈J. Then, there exists g∈G and h∈H with b≽g and b≽h. Hence, b≽gh, so $b\in \langle g_{1}h_{1},\ldots ,g_{n}h_{m}\rangle$. Conversely, if $b\in \langle g_{1}h_{1},\ldots ,g_{n}h_{m}\rangle$, then there exists a generator gh of $\langle g_{1}h_{1},\ldots ,g_{n}h_{m}\rangle$ with b≽gh. Since b≽gh, we must have b≽g and b≽h, so h∈I and h∈J, so h∈I∩J. □

**Proof of Lemma 3.** By the definition of content, we must have that $\Delta X_{i}$ is an ideal. Moreover, the intersection of ideals is an ideal, so the co-information $I(X_{1};\ldots ;X_{t})$ must also correspond to an ideal. □

**Proof of Theorem 3.** Firstly, we note that all co-informations correspond to ideals by the previous result in Lemma 3. It suffices to show that all ideals in ΔΩ correspond to some co-information. As such, given an ideal *J*, we need to find a collection of variables $X_{1},\ldots ,X_{t}$ where $I\left(X_{1};\ldots ;X_{t}\right)=\mu \left(J\right)$. Ideals are unique up to their generators, and we only need to consider sets of generators which are not contained in each other; otherwise, one of them is not needed as a generator. For each generator $g_{i}$, let $n_{i}=\deg g_{i}$. Let $S_{i}\subseteq \Omega$ be the set of elements in $g_{i}$. Then, consider the $2^{n_{i}}-2$ variables given by the partitions

(A1) $$X_{ij}=\left\{\left(\Omega \setminus S_{i}\right)\cup Q_{ij},S_{i}\setminus Q_{ij}\right\}$$

for every $Q_{ij}\subset S_{i}$ a non-empty proper subset of $S_{i}$ with $j\in \{1,\ldots ,2^{n_{i}}-2\}$. Intuitively, these variables spread the elements in $g_{i}$ across two partitions in every combination possible, so that the only guaranteed boundary crosses consistent across the entire collection $X_{ij}$ are by the $g_{i}$ atom and atoms in $\langle g_{i}\rangle$. Then

(A2) $$\underset{⌀\;\subset \;Q_{ij}\;\subset \;S_{i}}{⋂}\Delta X_{ij}=\langle g_{i}\rangle .$$

Here, we write $Q_{ij}$ to symbolise that these partitions are taken to obtain atom $g_{i}$. Across the set of generators $g_{1},\ldots ,g_{k}$, we consider all possible products

(A3) $$Y_{j_{1}j_{2}\cdots j_{k}}=X_{1j_{1}}\wedge X_{2j_{2}}\wedge \cdots \wedge X_{kj_{k}}=\underset{1\leq i\leq k}{⋀}X_{ij_{i}}.$$

where any combination from the $j_{i}$ where $1\leq j_{i}\leq 2^{n_{i}}-2$ can be taken. Note that we write A∧B to mean the coarsest partition which is finer than *A* and *B*. In practice, every variable corresponds to choosing one of the $Q_{j}$ for each generator, so every single combination is represented as a variable. Then

(A4) $$\underset{j_{1},\ldots ,j_{k}}{⋂}\Delta X_{j_{1}j_{2}\ldots j_{k}}=\langle g_{1},\ldots ,g_{k}\rangle$$

exactly, as any other generators will be removed, giving the result. □

**Proof of Corollary 2.** Given any atom *b*, we can consider the ideal I=〈b〉, and then using conditioning (in the sense of a set difference of information), we may subtract the higher co-information $C=\{{⋃}_{\omega \neq b}\langle b\omega \rangle \}$, which is itself an ideal, as the unions of ideals in lattices are ideals. We have that C⊆I, meaning we may condition it out in order to obtain the expression I∖C={b}. That is, *b* alone populates some region on an *I*-diagram between all variables $X_{a}$. Taking any collection of atoms hence corresponds to a collection of regions on the maximal *I*-diagram provided they are not counted with multiplicity. □

**Proof of Corollary 3.** There are $2^{n}-n-1$ elements in ΔΩ, as the points and the empty set do not contribute to the entropy, leaving $2^{n}-n-1$ atoms, and hence $2^{2^{n}-n-1}$ possible entropic expressions without multiplicity, including the zero expression. □

**Proof of Theorem 4.** Precisely those atoms ΔX are all those atoms which cross a boundary in *X*, that is, they must contain the pair $\omega_{a}\omega_{b}$ for $\omega_{a}\in P_{a}$ and $\omega_{b}\in P_{b}$ where $P_{a}$ and $P_{b}$ are different parts in the partition. The atom $\omega_{a}\omega_{b}$ can be written as the intersection of these two prime ideals. Since the union of ideals $I_{1}$ and $I_{2}$ is the ideal generated by the union of their generators in lattices, we have that ΔX must be the union across these parts and hence generated by 2-atoms. The second expression follows quickly from the first; every atom in $Q_{a}^{c}$ must lie in some other part $Q_{b}$ and vice versa. □

**Proof of Corollary 4.** All other atoms are described by whether or not they are contained in the intersection of the variable ideals $\Delta X_{a}$, which by the previous result are generated by degree 2 atoms. Hence, the knowledge of how these are distributed will describe the distribution of all other atoms. □

**Proof of Theorem 5.** We know that ΔX and ΔY are both degree 2 ideals as they are given by the union of intersections of prime degree 1 ideals, so their intersection ΔX∩ΔY can have generators of at most degree 4. Hence, we need to demonstrate that for every degree 3 or degree 4 generator in ΔX∩ΔY, there is a degree 2 generator which contains it. We demonstrate that every degree 4 atom in ΔX∩ΔY is contained in a degree 2 ideal. The argument for the degree 3 atoms is very straightforward and uses the same trick. Suppose that we have a degree 4 atom $\omega_{1}\omega_{2}\omega_{3}\omega_{4}$ which crosses a boundary in *X* and in *Y*. We may restrict to just these four outcomes $\omega_{1},\omega_{2},\omega_{3},\omega_{4}$, on which the partition of *X* and the partition of *Y* must now also restrict to a partition. Since $\omega_{1}\omega_{2}\omega_{3}\omega_{4}$ is contained in $\Delta X_{\left\{1,2,3,4\right\}}$ and $\Delta Y_{\left\{1,2,3,4\right\}}$, we must have that the local partition of *X* and the local partition of *Y* are non-trivial so that $\omega_{1}\omega_{2}\omega_{3}\omega_{4}$ crosses a boundary in this partition. Without loss of generality, the potential local partitions of any random variable $\Delta Q_{\left\{1,2,3,4\right\}}$ up to reordering of the $\omega_{i}$ are given by

(A5) $$\begin{array}{cc} \; & \langle 12,13,14\rangle \\ \; & \langle 13,23,14,24\rangle \\ \; & \langle 12,13,14,23,24\rangle \\ \; & \langle 12,13,14,23,24,34\rangle \end{array}$$

In particular, the total number of possible degree 2 generators in four outcomes is C24=6, so taking the intersection of $\Delta X_{\left\{1,2,3,4\right\}}\cap \Delta Y_{\left\{1,2,3,4\right\}}$ will, by the pigeonhole principle, have a degree 2 atom in their intersection unless both $\Delta X_{\left\{1,2,3,4\right\}}$ and $\Delta Y_{\left\{1,2,3,4\right\}}$ have at most three degree 2-atoms. Of the four possibilities above, only the first satisfies this possibility, so both *X* and *Y* are of this form. Without loss of generality, we assume $\Delta X_{\left\{1,2,3,4\right\}}$ is given by 〈12,13,14〉. The only possible degree 2 ideal which does not intersect with 〈12,13,14〉 is given by 〈23,24,34〉, so we should expect that $\Delta Y_{1,2,3,4}=\langle 23,24,34\rangle$. However, this does not correspond to any partition on {1,2,3,4}, as it does not contain a generator containing element 1. Thus, *Y* cannot have this form, and we must have that $\Delta X_{\left\{1,2,3,4\right\}}\cap \Delta Y_{\left\{1,2,3,4\right\}}$ must intersect and contain a degree 2 element, so the degree 4 atom $\omega_{1}\omega_{2}\omega_{3}\omega_{4}$ is contained in a degree 2 ideal. For any degree 3 atom, the argument is even simpler; the smallest possible ideal $\Delta X_{\left\{1,2,3\right\}}$ must have either 2 or 0 generators of degree 2 when restricted. If it had 0 generators, then {1,2,3} cannot cross a boundary in *X*, so it would not be present in ΔX∩ΔY. Hence, we must have $\Delta X_{\left\{1,2,3\right\}}$, which has at least two generators of degree 2 with the same being true for *Y*. As such, they must intersect with each other at a degree 2 atom by the pigeonhole principle, as the total number of possible generators is C23=3. □

**Proof of Corollary 5.** Using a result from [10], we have that $C_{\mathrm{GK}}\left(X;Y\right)=\mathrm{Rep}\left(\Delta X\cap \Delta Y\right)$ (the maximally representable subset inside of ΔX∩ΔY). We have now also shown that both ΔX∩ΔY and Rep(ΔX∩ΔY) are degree 2 ideals. Hence, the generators of the representable subset must be a subset of the generators of the mutual information. □

**Proof of Theorem 6.** We proceed by induction on *M* by showing that the theoretical minimum number of generators must still be large enough to force an overlap. We have demonstrated in the previous theorem that the statement is true for M=2. Suppose that the statement is true for M−1, then the ideal corresponding to the co-information $I(X_{1};\ldots ;X_{M-1})$ for the first M−1 variables has generators of degree at most M−1. Multiplying the generators of $\Delta I(X_{1};\ldots ;X_{M-1})$ by the generators for $\Delta X_{M}$, we hence know that $\Delta I(X_{1};\ldots ;X_{M})$ can be generated by atoms of at most degree M+1. Hence, we need to show that any degree M+1 atom is actually contained in a degree *M* ideal. We will use a similar counting argument to result Theorem 5. In particular, given a finite set of size *k* and two subsets of size $a_{1}$ and $a_{2}$, the minimum size of their intersection is given by $a_{1}+a_{2}-k$. Given three subsets, a minimum size for the intersection is then given by $\left(a_{1}+a_{2}-k\right)+a_{3}-k$ and so on. Hence, given *l* subsets, the corresponding expression is

(A6) $$a_{1}+\ldots +a_{l}-k\left(l-1\right).$$

Suppose that $\omega_{1}\ldots \omega_{M+1}$ is a degree M+1 atom contained in the co-information $\Delta I(X_{1},\ldots ,X_{M})$, which we need to demonstrate is contained in a degree *M* ideal. Restricting to $\left\{\omega_{1},\ldots ,\omega_{M+1}\right\}$, the minimum number of degree *M* atoms in $\Delta X_{i,\{1,\ldots ,M+1\}}$ for any *i* is given when $X_{i}$ corresponds locally to a partition of the form $\left\{\left\{\omega_{i}\right\},{\left\{\omega_{i}\right\}}^{c}\right\}$ for some single $\omega_{i}\in \Omega$ [If this is not immediately clear, consider any partition of Ω—we could choose a coarser sub-partition into two parts which must contain fewer 2-atoms, so minimising the number of 2-atoms overall is equivalent to finding the minimum number of degree 2 atoms in a partition of Ω into 2 parts. This is equivalent to minimising the value of $k\cdot \left(|\Omega |-k\right)=k|\Omega |-k^{2}$ for 0<k<|Ω|, which happens at k=1 or k=|Ω|−1]. Hence, there must be a minimum of CM−1M=M degree *M* atoms in $\Delta X_{i,\{1,\ldots ,M+1\}}$ (as we have already selected one outcome from the M+1 available outcomes—now we must select the other M−1 outcomes). The maximum size of the set of all possible degree *M* atoms in the restriction to {1,…,M+1} is CMM+1=M+1. Hence, taking the intersection of *M* variables, assuming a minimal number of degree *M* atoms, and using the expression in Equation (A6), we need to only to demonstrate that

(A7) M·M−(M−1)·(M+1)>0,

which always evaluates to unity, proving that there is at least one degree *M* ideal containing every degree M+1 atom in the intersection, proving the result. □

**Proof of Theorem 4.** Let our ideal be $\langle \omega_{1}\ldots \omega_{k}\rangle$. We will proceed by induction on the difference d=|Ω|−k, arguing at each step that the upper set is monotonic in probability of the last element $\omega_{k+d}$. We note that the sign of the upper set might only change if it contains additional outcomes, so provided we treat this carefully, we can also allow Ω to vary (via restriction) provided that it always contains $\omega_{1},\ldots ,\omega_{k}$. As earlier, we will write ${\langle \omega_{1}\ldots \omega_{k}\rangle }_{S}$ to illustrate that we are operating inside some restricting set *S*. These quasi-ideals are quite justified, as all of the previous results must still hold even if we assume the probabilities inside of *S* do not sum to 1. These atoms will still have the same measure regardless of the context *S* in which we find them. For the first case |Ω|=k, we note that ${\langle \omega_{1}\ldots \omega_{k}\rangle }_{\left\{\omega_{1},\ldots ,\omega_{k}\right\}}$ consists of the single atom $\omega_{1}\ldots \omega_{k}$. We will use the shorthand notation ${\langle \omega_{1},\ldots ,\omega_{k}\rangle }_{\left\{\omega_{1},\ldots ,\omega_{n}\right\}}={\langle \omega_{1},\ldots ,\omega_{k}\rangle }_{n}$ for some simplicity. By Theorem 1, which characterises the sign of individual atoms, we have both that ${\left(-1\right)}^{k}\mu \left({\langle \omega_{1}\ldots \omega_{k}\rangle }_{k}\right)>0$ for $P(\omega_{k})\neq 0$ and that $\mu ({\langle \omega_{1}\ldots \omega_{k}\rangle }_{k})$ varies monotonically in $P(\omega_{k})$ between 0 and $-\mu (\omega_{1},\ldots ,\omega_{k-1})$. So, the theorem is true for d=0. Now, suppose that $\mu ({\langle \omega_{1}\ldots \omega_{k}\rangle }_{k+d})$ varies monotonically in $P(\omega_{k+d})$ between 0 and $-\mu ({\langle \omega_{1}\ldots \omega_{k}\rangle }_{k+d-1})$. Then, we first note that

(A8) $$\begin{array}{c} \begin{array}{c} {\langle \omega_{1}\ldots \omega_{k}\rangle }_{\left\{\omega_{1},\ldots ,\omega_{k+d},\omega_{k+d+1}\right\}}={\langle \omega_{1}\ldots \omega_{k}\rangle }_{\left\{\omega_{1},\ldots ,\omega_{k+d}\right\}}\\ \cup \;\;\omega_{k+d+1}\;{\langle \omega_{1}\ldots \omega_{k}\rangle }_{\left\{\omega_{1},\ldots ,\omega_{k+d}\right\}}. \end{array} \end{array}$$

where we use the multiplicative notation to signify that $\omega_{k+d+1}$ is added as an outcome to all atoms in $\langle \omega_{1}\ldots \omega_{k}\rangle$. For example,

(A9) $$4\cdot {\langle 12\rangle }_{\left\{1,2,3\right\}}=\left\{124,1234\right\}.$$

Hence, we can view $\mu (\langle \omega_{1}\ldots \omega_{k}\rangle )$ as a function on $P\left(\omega_{k+d+1}\right)=p_{k+d+1}$:

(A10) $$\begin{array}{c} \begin{array}{c} \mu \left({\langle \omega_{1}\ldots \omega_{k}\rangle }_{\left\{\omega_{1},\ldots ,\omega_{k+d+1}\right\}}\right)=\mu \left({\langle \omega_{1}\ldots \omega_{k}\rangle }_{\left\{\omega_{1},\ldots ,\omega_{k+d}\right\}}\right)\\ +\mu (\omega_{k+d+1}{\langle \omega_{1}\ldots \omega_{k}\rangle }_{\left\{\omega_{1},\ldots ,\omega_{k+d}\right\}}) \end{array} \end{array}$$

We now notice that the second term can be expressed

(A11) $$\begin{array}{c} \begin{array}{c} \mu (\omega_{k+d+1}{\langle \omega_{1}\ldots \omega_{k}\rangle }_{\left\{\omega_{1},\ldots ,\omega_{k+d}\right\}})\\ =\mu ({\langle \omega_{1}\ldots \omega_{k}\omega_{k+d+1}\rangle }_{\left\{\omega_{1},\ldots ,\omega_{k},\omega_{k+d+1}\right\}}) \end{array} \end{array}$$

But now we can see that the difference between k+1 and k+d+1 is just *d*, so this reduces to the case for *d*. By assumption, we hence have that this ideal varies monotonically on $\omega_{k+d+1}$ between 0 and $\mu ({\langle \omega_{1}\ldots \omega_{k}\rangle }_{k+d})$. This means that the entire expression in Equation (A11) must monotonically vary between 0 and $\mu (\langle \omega_{1}\cdots \omega_{k}\rangle )$ as a function of $\omega_{k+d+1}$. Since we can construct each ideal by successively increasing *d* and this leaves the sign intact (note that no probability tends to infinity), the sign is left unchanged, proving the result. □

**Proof of Theorem 7.** Let $J=\langle g_{1},\ldots ,g_{j}\rangle$ be an ideal of strong even/positive parity with the result for odd/negative parity following equivalently. By the definition of strong even parity, we must have

(A12) $$\mu \left(J\right)=\sum_{\alpha \in A}P\left(J_{\alpha }\right)\mu \left(J_{\alpha }\right).$$

Every strong fixed-parity ideal is defined in terms of an a finite sum of ideals with one generator, so we may assume without loss of generality that the $J_{\alpha }$ has single generators. By virtue of Lemma 4, we know that $P\left(J_{\alpha }\right)=\mathrm{sgn}\left(\mu \left(J_{\alpha }\right)\right)$. Hence, taking the sum across all the $J_{\alpha }$ values, we have that all of the terms $P\left(J_{\alpha }\right)\mu \left(J_{\alpha }\right)$ must be positive. As all terms in the sum positive, so too is μ(J). □

**Proof of Theorem 8.** We will first allow ourselves to consider probabilities not summing to one, demonstrating that the sign has a given parity, and then we shall scale appropriately using the homogeneity property of μ to obtain meaningful probabilities once more, while the parity shall be fixed. Suppose *J* is a strongly mixed ideal. Then, *J* has an even degree generator *g*. We first send all atoms in Ω∖g (as a set) to 0. Then, we have

(A13) μ〈J〉=μ〈g〉>0.

Summing the probabilities, we let $K=\sum_{\omega \in g}P\left(\omega \right)$. Then, we scale

(A14) $$0\leq \frac{1}{K}\mu \left(\left\{P\left(\omega \right):\omega \in g\right\}\right)=\mu \left(\left\{\frac{P(\omega )}{K}:\omega \in g\right\}\right)$$

where we now have $\sum_{\omega \in g}\frac{P(\omega )}{K}=1$. Hence, we have found a given set of probabilities where μ(J)>0. Repeating the exercise for *g* of odd degree will similarly show that there are probabilities such that μ(J)<0. Hence, μ(J) can be either positive or negative given a strongly mixed-parity ideal, giving the result. □

**Proof of Theorem 9.** We begin by briefly demonstrating that Z=XOR(X,Y) has co-information ΔI(X;Y;Z) given by a strongly odd parity ideal. Given the outcomes

*X*	*Y*	*Z*	ω
0	0	0	1
0	1	1	2
1	0	1	3
1	1	0	4

we have that ΔX∩ΔY∩ΔZ=〈123,124,134,234〉. In this case, we have

(A15) $$\begin{array}{cc} \mu (\langle 123,124,134,234\rangle ) & =\mu (\langle 123,124\rangle )+\mu (\langle 134,234\rangle )\\ \; & \;-\mu (\langle 123,124\rangle \cap \langle 134,234\rangle )\\ \; & =\mu (\langle 123,124\rangle )+\mu (\langle 134,234\rangle )\\ \; & \;-\mu (\langle 1234\rangle ) \end{array}$$

where now we also have

(A16) $$\begin{array}{cc} \mu (\langle 123,124\rangle ) & =\mu (\langle 123\rangle )+\mu (\langle 124\rangle )-\mu (\langle 1234\rangle ) \end{array}$$

(A17) $$\begin{array}{cc} \mu (\langle 123,234\rangle ) & =\mu (\langle 123\rangle )+\mu (\langle 234\rangle )-\mu (\langle 1234\rangle ). \end{array}$$

Working backwards, we see that 〈123,124〉 and 〈134,234〉 are negative (odd) fixed-parity ideals, so that ΔX∩ΔY∩ΔZ in this case is a negative fixed-parity ideal. To show that there are no other such deterministic functions on three variables, we start by considering the case where *X* and *Y* are both binary variables. In this case, we know that we can express all events on *Z* in terms of f(X,Y) on the four outcomes

*X*	*Y*	ω
0	0	1
0	1	2
1	0	3
1	1	4

In this case, we have ΔX∩ΔY=〈14,23〉, which is known to have positive measure as it reflects a mutual information. Similarly, any subset 〈14〉 or 〈23〉 will also have a positive measure, so we cannot have an ideal generated by a degree 2 ideal alone. However, the ideal cannot have even and odd generators (as then it would have mixed parity by Theorem 8 and it cannot have generators more than degree 3 by Theorem 6). Hence, the ideal must be exclusively generated by degree 3 atoms and we must nullify these two degree 2 atoms. Hence, we know that in order to have degree 3 atoms generating I(X;Y;Z), *Z* must have equal values on the outcome pairs {1,4} and {2,3}. Moreover, we cannot have that f(0,0)=f(0,1)=f(1,0)=f(1,1) for all outcomes, as then I(X;Y;Z)=0. Hence, we must have that f(0,0)=f(1,1)=0 and f(0,1)=f(1,0)=1; that is, *Z* is the XOR gate. We now extend by induction to give the full result. Let $N_{X}$ be the number of events in *X* and $N_{Y}$ the total number of events in *Y*. In the case where either $N_{X}$ or $N_{Y}$ is 1, then that variable must be constant and have zero entropy, so the co-information I(X;Y;Z) is trivially zero. We have seen that in the case where $N_{X}=N_{Y}=2$ that the only negative fixed-parity system of the form X,Y,f(X,Y) is the XOR gate. We consider the case $N_{X}=3$ and $N_{Y}=2$ to highlight the inductive argument. In this case, again, we know that *Z* can be computed deterministically from *X* and *Y*, allowing us to use the same trick. Labelling outcomes, we have

*X*	*Y*	ω
0	0	1
0	1	2
1	0	3
1	1	4
2	0	5
2	1	6

In this case, we see that

(A18) ΔX∩ΔY=〈14,16,23,25,36,45〉.

Again, this is a mutual information and hence positive. Thus, we know that for *Z* to be purely negative, it must be generated by purely degree 3 atoms, so *Z* must remain unchanged on these pairs of outcomes. Assigning a symbol to Z(0,0), we can use the same trick as before and successively annihilate various pairs of atoms, giving us the following chain:

(A19) $$\begin{array}{cc} \; & Z(0,0)=0\\ \Rightarrow & Z(1,1)=0,\;Z(2,1)=0\\ \Rightarrow & Z(2,0)=0,\;Z(1,0)=0\\ \Rightarrow & Z(0,1)=0 \end{array}$$

That is to say, *Z* does not vary on Ω and the co-information is zero in this case. We now suppose for the induction that there are no fixed-parity systems with $N_{X}$ outcomes on *X* and $N_{Y}$ outcomes on *Y*. We will demonstrate that we can introduce an additional event to either *X* or *Y* and we will still obtain that *Z* is the trivial variable. Without loss of generality, we increase $N_{X}$ by one. This will introduce $N_{Y}$ additional outcomes. As we have (without reference to the probabilities) demonstrated that $Z(\omega_{1})=0$ for $\omega_{1}\in S_{1}=\left\{1,\ldots ,N_{X}N_{Y}\right\}$, it suffices to show that for every further outcome $\omega_{2}\in S_{2}=\left\{N_{X}N_{Y}+1,\ldots ,\left(N_{X}+1\right)N_{Y}\right\}$, there is an atom $\omega_{1}\omega_{2}$ with $\omega_{1}\in S_{1}$. For each $\omega_{2}$, we shall pick some $\omega_{1}\in S_{1}$ such that $\omega_{1}\omega_{2}\in \Delta X\cap \Delta Y$. Using the ordering we have utilised so far and restricting to the bottom of the table, we may assume that $X\left(\omega_{2}\right)=N_{X}$ as a symbol, and $Y\left(\omega_{1}\right)\in \left\{0,\ldots ,N_{Y}-1\right\}$. Given $\omega_{2}$, we may select the outcome $\omega_{1}$ to be the outcome corresponding to $X=X(\omega_{2})-1$ and $Y=Y(\omega_{2})-1$, where in *Y* we perform arithmetic mod $N_{Y}$. We must then have that *X* and *Y* both change when moving from $\omega_{1}$ to $\omega_{2}$. By the definition of content, this means that the $\omega_{1}\omega_{2}$ atom will be contained in ΔX∩ΔY, as needed, so we must have $Z\left(\omega_{2}\right)=Z\left(\omega_{1}\right)=0$, showing that *Z* is actually the trivial variable, inductively giving the result. □
